# Crush syndrome: a review for prehospital providers and emergency clinicians

**DOI:** 10.1186/s12967-023-04416-9

**Published:** 2023-08-31

**Authors:** Daisuke Usuda, Shintaro Shimozawa, Hiroki Takami, Yoshinobu Kako, Taigo Sakamoto, Junya Shimazaki, Junichi Inoue, Shinichi Nakayama, Yuichi Koido, Jiro Oba

**Affiliations:** 1 Department of Emergency and Critical Care Medicine, Juntendo University Nerima Hospital, 3-1-10, Takanodai, Nerima-City, Tokyo, 177-8521 Japan; 2 Department of Sport Management, Faculty of Business Informatics, Jobu University, 634-1, Toya-Chou, Isesaki-City, Gunma, 372-8588 Japan; 3 Department of Emergency and Critical Care Medicine, Nippon Medical School, Graduate School of Medicine, 1-1-5, Sendagi, Bunkyo-City, Tokyo, 113-8602 Japan; 4 Department of Traumatology and Acute Critical Medicine, Osaka University Graduate School, 2-15, Yamadaoka, Suita-City, Osaka, 565-0871 Japan; 5 Department of Emergency and Critical Care Medicine, Nippon Medical School Musashikosugi Hospital, 1-383, Kosugi-Cho, Nakahara-Ku, Kawasaki-City, Kanagawa, 211-8533 Japan; 6 Department of Emergency and Critical Care Medicine, Hyogo Emergency Medical Center, 1-3-1, Wakinohamakaigandori, Chuo-Ku, Kobe-City, Hyogo, 651-0073 Japan; 7 National Hospital Organization Headquarters, DMAT Secretariat MHLW Japan, 3256, Midoricho, Tachikawa-City, Tokyo, 190-8579 Japan

**Keywords:** Disaster, Crush syndrome, Crush injury, Ischemia reperfusion, Treatment, Outcome

## Abstract

**Introduction:**

Disasters and accidents have occurred with increasing frequency in recent years. Primary disasters have the potential to result in mass casualty events involving crush syndrome (CS) and other serious injuries. Prehospital providers and emergency clinicians stand on the front lines of these patients’ evaluation and treatment. However, the bulk of our current knowledge, derived from historical data, has remained unchanged for over ten years. In addition, no evidence-based treatment has been established to date.

**Objective:**

This narrative review aims to provide a focused overview of, and update on, CS for both prehospital providers and emergency clinicians.

**Discussion:**

CS is a severe systemic manifestation of trauma and ischemia involving soft tissue, principally skeletal muscle, due to prolonged crushing of tissues. Among earthquake survivors, the reported incidence of CS is 2–15%, and mortality is reported to be up to 48%. Patients with CS can develop cardiac failure, kidney dysfunction, shock, systemic inflammation, and sepsis. In addition, late presentations include life-threatening systemic effects such as hypovolemic shock, hyperkalemia, metabolic acidosis, and disseminated intravascular coagulation. Immediately beginning treatment is the single most important factor in reducing the mortality of disaster-situation CS. In order to reduce complications from CS, early, aggressive resuscitation is recommended in prehospital settings, ideally even before extrication. However, in large-scale natural disasters, it is difficult to diagnose CS, and to reach and start treatments such as continuous administration of massive amounts of fluid, diuresis, and hemodialysis, on time. This may lead to delayed diagnosis of, and high on-site mortality from, CS. To overcome these challenges, new diagnostic and therapeutic modalities in the CS animal model have recently been advanced.

**Conclusions:**

Patient outcomes can be optimized by ensuring that prehospital providers and emergency clinicians maintain a comprehensive understanding of CS. The field is poised to undergo significant advances in coming years, given recent developments in what is considered possible both technologically and surgically; this only serves to further emphasize the importance of the field, and the need for ongoing research.

**Supplementary Information:**

The online version contains supplementary material available at 10.1186/s12967-023-04416-9.

## Introduction

Each year, millions of people worldwide experience natural disasters (such as earthquakes, hurricanes or typhoons, flooding, and landslides) or human-made disasters (such as terrorist attacks, airplane or train crashes, and wars) [1]. At present, some 800 million people live in areas that are prone to earthquakes, or at high risk of severe tropical hurricanes or typhoons [1]. In recent years, disasters and accidents have been frequent occurrences [2]. Recent examples include the 2023 earthquake that struck Turkey and Syria, resulting in more than 47,000 deaths and displacing millions of people [1]. Serious injuries such as crush syndrome (CS) can commonly result from primary disasters such as these, whether the disasters are natural or human-made [1, 2]. CS is defined as the systemic manifestation of severe, traumatic muscle injury [1]. Prehospital providers and emergency clinicians stand on the front lines of these patients’ evaluation and treatment [1]. At present, the American College of Emergency Physicians and others acknowledge in literature that "crush injury (CI)" refers to tissue injury, while “CS” refers to crush injuries with systemic manifestations. This is noted as well by Bywaters and Beall, the physicians who initially described this medical condition in 1941 [2]. In addition, the bulk of current knowledge on the topic is based on historical data, remaining unchanged for over ten years [3]. To date, no evidence-based treatment has been established [2]. Therefore, there is a need to review and update this topic.

## Objective

This narrative review aims to provide a focused overview of CS, including etiology, mechanisms, symptoms, examinations, diagnosis, treatments, complications, prognoses, knowledge from actual disasters, and other future medical science concerns, for the benefit of prehospital providers and emergency clinicians.

## Discussion

### Crush injury

CI is caused by direct physical trauma and compression of the human body; the lower extremities are the most common site of CIs [1]. CIs occur in various settings, including motor vehicle accidents (both at home and elsewhere) and other types of accidents and disasters (including earthquakes, landslides, mine disasters, explosions, collapsing buildings, terrorist attacks, and wars), and they represent a spectrum of bodily injuries as caused by trauma [2, 4–6]. Typically, these injuries involve multiple types of tissue, including skin and subcutaneous tissue, muscle and tendon tissue, and/or bone and joint tissue [5]. Even when CIs do not involve vital organs, they can still be life-threatening, especially when suffered in the extremities [4]. CI may result in asphyxia, severe orthopedic injury, compartment syndrome, hypotension, and organ injury (including acute kidney injury (AKI)) [1]. CIs in adults have been well studied and documented, and a United States-based cohort study found that CIs were correlated with a significant increase in the likelihood of acute compartment syndrome (ACS) (odds ratio of 1.83) [7]. On the other hand, CIs rarely occur among the pediatric population [8]. Details regarding CIs for each part of the body are as discussed below.

Chest CIs often necessitate rapid intervention and stabilization, with early diagnosis and surgical repair being crucial when it comes to minimizing complications and loss of respiratory function in the patient [1]. CIs to the upper chest in particular may lead to acute traumatic trachea disruption [1]. One Chinese study investigating the pathogenesis, diagnosis, and treatment of CSs of the chest and arm found that factors leading to severe, rapid CS progress include carbon monoxide poisoning, drunkenness, and one’s own body parts being pressed together [9]. Key to treatment is comprehensive therapy, including thorough and rapid reduction in tension to preserve limb function, continuous renal replacement therapy, and corrections to anemia and electrolyte imbalance [9].

Severe abdominal CIs have the potential to affect various organ systems, including the musculoskeletal, urological, cardiovascular, integumentary, and digestive systems [10]. Any system damaged by a severe abdominal CI has the potential to for fatal outcomes [10]. One previously published study indicated a survival rate of only 10.6% in traumatic cardiac arrest patients transported to UK hospitals in Afghanistan and Iraq [10]. CSs and AKIs have also been reported to be two major causes of death following earthquakes [10]. Furthermore, mesenteric laceration can cause severe bleeding, which in turn can result in hemorrhagic shock and multiple organ failure [10]. Fractures caused by abdominal CIs may cause arterial hemorrhaging, which itself can result in hemorrhagic shock [10].

Pelvic vascular injuries are ordinarily the result of high-energy trauma [11, 12]. Most of these injuries are the result of motor vehicle collisions, with the rest due to causes such as falls or industrial CIs [11]. Pelvic vascular injuries have a frequent association with disruption of the pelvic ring, and incur high mortality rates due to shock caused by pelvic bleeding [11]. The morbidity and mortality that result from pelvic vascular injuries are due to pelvic hemorrhage and the exsanguination that results; this can potentially be treated or even reversed if diagnosed early using multidetector computed tomography (CT) and promptly treated [11].

### Crush syndrome

The term “Crush syndrome” is generally used to refer to the destruction of muscle tissue after direct trauma, injury, or compression, and presents at times in advanced stages due to the amount of time needed to locate and extricate those who suffer from it; it is also a severe systemic manifestation of trauma and ischemia that involves soft tissue, primarily skeletal muscle, due to the prolonged crushing experienced by the tissue [3, 13–15]. It was first observed during the 1901 Messina earthquake in Italy, and was first described by Bywaters et al. in 1941, when they noted the relationship between muscle necrosis and a brown pigment that was found during autopsies in the renal tubules of patients who had been buried for several hours by bombings of London during World War II [2, 3]. To this day, the majority of cases of CS-associated rhabdomyolysis that develop life-threatening complications are due to both natural and human-made disasters [2, 3]. This potentially lethal condition is characterized by muscle cell damage as a result of the decompression that follows compression (i.e. ischemia reperfusion (IR) injuries) [16, 17]. After direct trauma, CS is the second most common cause of death due to earthquakes [18]. The onset can occur as soon as an hour post-injury [6]. According to a systematic review, the reported CS incidence rate among earthquake survivors was 2–15% [19]. In addition, survival required limiting the degree of renal dysfunction and supporting organ function, and mortality was reportedly up to 48% [19].

### Etiology, mechanism, and symptoms

A compressive force crushes a portion of the body, transiently increasing the pressures within [20]. This force acts on the incompressible vasculature blood, dramatically increasing tissue pressures and causing damage to multiple types of tissue, including bones, blood vessels, nerves, and soft tissue [20]. A broad zone of injury can result from a delayed inflammatory reaction involving the zone around the crushed cells, which may obscure the injury’s severity at first [20]. Pressure within the skeletal muscle compartment can be increased by edema and/or bleeding within the fascial envelope’s confines [5]. When the fluid pressure of the tissue within the compartment becomes greater than the muscle and nerve capillary perfusion pressure in the compartment, these tissues become ischemic, and begin to show signs and symptoms of skeletal muscle-compartment syndrome [5]. Coagulation of lymph in the development of tissue pathological conditions has been shown to potentially outpace the changes that can be observed in the blood [21]. As such, these injuries have a tendency to cause a great deal of inflammation and swelling, potentially followed by compartment syndrome, or another form of vascular damage, infection, neurological injury, and/or tissue necrosis [20]. Though it was believed that skeletal muscle was relatively ischemia-tolerant for a period of 2–4 h without permanent injury, irreversible changes that can limit functional recovery likely start to occur in as little time as 1 h, especially in the event of concurrent tissue damage and/or other injuries [19].

IR injuries occur in the event of tissue re-perfusion following an ischemic period, as a result of acute inflammation that can involve various mechanisms [22]. This intense inflammatory response has both local and systemic effects as a result of the physiological, biochemical, and immunological changes that happen during the periods of ischemia and reperfusion [22]. Though it is generally not caused solely by the accumulation of free blood or fluid in the compartment, in some cases, this accumulation can be a contributing factor [23]. The pathophysiology resolves around a self-perpetuating edema-and-ischemia cycle, with severity spanning a gamut from mild to the point of near non-existence, all the way to tissue death [24].

Systemic involvement of CI is referred to as CS, and occurs as a result of tissue ischemia and muscle necrosis [25]. Prolonged muscle ischemia increases the permeability of cell membranes, and causes cells to release potassium, enzymes, and myoglobin [13]. As a result of the severe damage and swelling to the muscle tissue due to IR, compartment syndrome becomes nearly inevitable [26].

As blood re-perfuses into the ischemic muscle, an immediate inflammatory response is triggered, and neutrophils are the first to infiltrate, exacerbating the damage to the muscle [27]. CS — the systemic manifestation of muscle cell breakdown, with contents being released into circulation — leads to metabolic derangement and AKI [4]. In particular, a major pathological manifestation of CS-AKI is dysfunction of renal tubular epithelial cells and cell death, attributed to large-scale myoglobin deposition [15]. Large quantities of myoglobin, released by damaged muscle, deposit within the renal tubules and impede their proper function, directly damaging the tubules through elevated levels of iron and oxidative stress [15]. The characteristic features of ferroptosis are iron overload and lipid peroxidation damage [15]. Additionally, ferroptosis has been demonstrated to be promoted by high levels in renal tissue of pro-inflammatory cytokines and damage-associated molecule pattern molecules (HMGB1, double-strand DNA, and macrophage extracellular trap) [15]. However, it remains unclear what the mechanism of ferroptosis is in CS-AKI, as well as whether it could serve as a therapeutic target [15]. Combined with systemic hypotension, this can result in renal dysfunction with acute tubular necrosis and uremia [13].

**Fig. 1:**
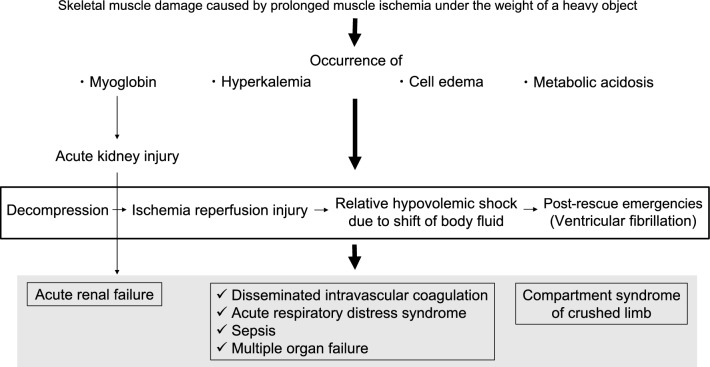
Mechanism by which CS occurs. Skeletal muscle damage is caused by prolonged muscle ischemia, under the weight of a heavy object. IR injuries caused by decompression cause various CS conditions

CS patients have been known to develop cardiac failure, kidney dysfunction, shock, systemic inflammation, and sepsis [16]. In addition, late presentations can include life-threatening systemic effects, including hypovolemic shock, hyperkalemia, and metabolic acidosis, and very late cases can present as disseminated intravascular coagulation [13, 28].  The above-mentioned mechanism, and the symptoms by which CS occurs, are shown in Fig. 1.

### Diagnosis

At present, diagnosis of ACS is made on the basis of physical examination, as well as measurement of intra-compartmental pressure through repeated needle-pricks over a short time frame [23, 29]. A threshold value of < 20 mmHg difference between the intra-compartmental pressure and diastolic blood pressure is considered to serve as a diagnostic [29]. However, diagnosis of compartment syndrome of the foot remains challenging because the signs and symptoms remain less reliable indicators compared to those for compartment syndrome elsewhere in the body [23, 30]. In addition, current continuous pressure measurement technology lacks the necessary sensitivity, particularly in deep tissues and compartments, and use of this technology is limited to highly trained personnel [23]. Therefore, diagnosing ACS is particularly difficult in disaster-stricken areas, which can lead to delayed diagnoses [2, 30].

### Treatment

The most important measure available to reduce CS mortality in disaster situations is the immediate start of treatment [2]. ACS in particular can be a devastating condition: if not treated in a timely fashion, it is associated with lasting consequences, or even death [8, 31]. Care at the scene of the incident is essential, with a focus on the treatment of life-threatening injuries, as well as extrication, triage, fluid resuscitation, and transport [1]. Healthcare facility care includes initial stabilization and evaluation of trauma, as well as treatment of any complications (such as hyperkalemia, compartment syndrome, rhabdomyolysis, or AKI) [1].

In large-scale natural disaster areas, timely access to treatment equipment, for treatments such as continuous administration of large amounts of fluid, diuresis, and hemodialysis, becomes unfeasible [2, 32]. To reduce CS complications, early, aggressive resuscitation in prehospital settings, ideally even before extrication, is recommended [4]. Providers must be aware of hyperkalemia risk shortly after extrication; the mainstay treatment is ongoing resuscitation with intravenous fluids [4]. For crush victim treatment, it is crucial to emphasize the importance of early fluid administration, even before victim extrication, and avoiding solutions that contain potassium [18]. Ideally, intravenous fluids are to be initiated as soon as possible (preferably within 6 h of the muscle injury), at a rate that maintains an adult urine output of at least 300 mL/h, for a minimum of the first 24 h [33]. Sodium bicarbonate (NaHCO_3_) should be administered only when necessary for the correction of systemic acidosis and mannitol, and only to maintain urine output of at least 300 mL/h despite the administration of adequate fluids [33]. In addition, rapid fluid therapy accompanied by prophylactic CI cocktail administration (namely mannitol-bicarbonate), by mixing 40 mEq NaHCO_3_ and 50 ml of 20% mannitol into 1,000 ml of 0.45% NaCl and 5% dextrose, was found to be largely effective in preventing acute renal failure (ARF) from developing in cases with disaster-caused CS [14]. To date, however, no randomized controlled trials have compared intravenous fluid therapy with bicarbonate and/or mannitol versus intravenous fluid therapy alone [3]. In addition, some evidence has been established regarding prophylaxis of CS, but these treatments’ efficacy has yet to be clearly determined [34].

Icing therapy performed over the affected muscle is reportedly effective for improvements to mitochondrial dysfunction and inflammation [35]. These effects are believed to be secondary to improvements in early-disease-stage leakage of potassium and myoglobin from damaged myocytes [35]. Icing therapy serves to temporarily prolong viability after CI [35]. Combining CI with other infusion therapies can improve its efficacy [35].

Because ischemia is a core component of traumatic ischemia, and hypoxia occurs as a consequence of ischemia, hyperbaric oxygen logically serves as an intervention for these conditions in situations that pose threats to tissue survival, infection control, and healing [24]. In these cases, oxygen is considered a drug, with its own contraindications and adverse effects; hyperbaric oxygen therapy (HBOT) has been approved for use as primary or adjunctive care [36, 37]. Particularly in incipient stages, before fasciotomy is necessary, treatment presents a therapeutic challenge, because there are no means to arrest progression other than HBOT [5]. A “hyperbaric” environment is one in which a patient's entire body is physically exposed to 100% oxygen, at a pressure greater than 1 atmosphere (absolute) [37]. HBOT works through ideal gas laws, and is effective in treating CIs as an adjunctive therapy [37]. HBOT works on CIs through various mechanisms [37]. The effects of hyperoxygenation, edema reductions, enhanced infection control, formation of blood vessels and collagen, and the reduction of free radicals and reperfusion injuries assist CI patients’ healing [37]. The benefits of HBOT include tissue repair, and indications comprise a broad range of diseases, ranging from intoxication to IR injuries, including CS [36, 38]. Unfortunately, even with hyperbaric oxygen mechanisms that strongly support its use in traumatic ischemia, coupled with supporting clinical data, clinicians are generally disinclined toward its use for such conditions [5, 24]. One of the most significant obstacles preventing acceptance is surgeon bias [36]. This is because strong arguments exist for its use, based on evidence-based data, and based on how HBOT mitigates the conditions’ pathology [5].

The delayed onset of reperfusion injury through commercial tourniquet use has been previously described in theory [34]. There is now extensive literature that supports the safety of using a tourniquet in limb trauma cases, and further study is merited of this potentially life-saving measure in CI patients [34]. The application of a tourniquet prior to extrication is not broadly recommended in order to prevent CS; it is therefore strongly recommended in prehospital settings for severe CIs [25, 39, 40]. There is also a reported case of prehospital tourniquet application in order to delay reperfusion injury after CI, resulting in reduced morbidity and complete limb salvage [34]. When it is possible to transfer to a higher level of care, tourniquet application to the crushed limb could serve to prevent loss of life from the hemorrhaging or electrolyte abnormalities secondary to CS [13]. However, this remains controversial [13].

A fasciotomy is an emergency procedure used to treat ACS, occurring most often in the volar compartment of the forearm, the deep posterior, or the anterior compartment of the leg. It is the only known effective treatment for ACS following a fracture, and this procedure is performed in 0.7% of all foot fractures [26, 41]. One study found that open fractures, CIs, and multiple foot fractures served as the strongest fasciotomy predictors [3]. However, controversy surrounds the role of fasciotomies in the treatment of CS caused by CIs [42]. There is no clear evidence that fasciotomy actually improves outcomes; rather, delayed fasciotomy was reported to have resulted in worse physical outcomes [26, 30]. Nevertheless, an emergent fasciotomy is a common recommendation in order to try to prevent chronic pain and deformities [30]. Clinicians should consequently maintain a very low fasciotomy threshold, to prevent long-term sequelae that are associated with undiagnosed compartment syndrome [8]. On the other hand, fasciotomies for CI have relatively frequent complications, with the most serious of these being uncontrolled bleeding and sepsis due to infections of the wound [26]. Reports from Iran and Turkey similarly show associations between fasciotomy and sepsis and sepsis-related mortality [26]. For open wounds, however, cleaning and debridement are vital [26]. On the other hand, there appears to be no dispute that, in cases where the peripheral pulses of the extremities are not palpable, surgical decompression is required [26].

Surgical intervention may also be necessary to correct secondary deformities [30]. In particular, CIs with minimal skin disruptions can prove especially difficult to manage [20]. The main objectives of severe CIs treatment are debriding away devitalized tissue, and filling in any resulting dead space with vascularized tissue [43, 44]. One article reports that the optimal methods for soft tissue coverage in CI treatment are the iliac flap, the adipofascial lateral arm flap, and the gracilis flap [43, 44]. The accompanying bone defects have been found to respond very positively to free corticoperiosteal flaps [43, 44]. Digital defects often require complete or subtotal toe transfer in order to avoid amputation and restore hand function [43, 44].

The decisions involved in choosing amputation, as opposed to limb salvage of the upper limb, are multifactorial [45]. For limb salvage, the key factors to consider can be categorized into three main subgroups: global patient factors, limb-specific factors, and injury mechanism factors, with details shown in Table 1 [45]. The primary global patient factor is uncontrollable hemodynamic instability [45]. If vital signs and symptoms continue to worsen despite aggressive resuscitation, amputation may be necessary [26]. Limb-specific factors include extensive and concurrent soft tissue, bone, vascular, and/or nerve injuries; and prolonged limb ischemia [45]. Injury mechanism factors include blunt arterial trauma and CIs [45]. Amputation was generally performed when elements from at least two of these subgroups were present [45]. The following scoring systems are currently recommended for aiding decisions regarding amputation: the Mangled Extremity Severity Score, and the Mangled Extremity Syndrome Index [45]. However, the existing scoring systems are built predominantly on lower limb trauma, and lack robust evidence to guide upper extremity management [45, 46]. Validating scoring systems that could aid in decision-making, and provide further outcome information from the two treatment options, would require further high-quality studies [45]. It also remains unclear whether early amputation has patient-centered benefits [42]. On the other hand, risk factors for poor prognosis can suggest amputation might be favored, despite apparent limb viability and the morbidity of extremity loss [42].

**Table 1:** Key factors when considering amputations for CS

Factor	Details
Global patient	Presence of uncontrollable hemodynamic instability
Limb-specific	Extensive and concurrent soft tissue, bone, vascular and/or nerve injuries
	Prolonged limb ischemia
Mechanism of injury	Blunt arterial trauma
	CIs

The key factors are categorized into three subgroups: global patient factors, limb-specific factors, and injury mechanism factors, and details are shown for each. Amputation is generally performed when there are elements from at least two of these subgroups present

### Complications and prognosis

Complications arising from the CI give rise to CS, and the sequelae of the IR cellular injury can lead to lost organ or limb function, or even death [2, 22]. CS causes ARF secondary to hypovolemia and traumatic rhabdomyolysis, hyperkalemia, electrolyte abnormalities, arrhythmia, and metabolic acidosis from muscle damaged at any of three different times: at the time of initial mechanical crushing force, during periods of ischemia, and during reperfusion [19, 26, 32, 47]. These occur especially in patients who have extended treatment and extrication times [4, 6]. Of these, hyperkalemia and ARF represent life-threatening CI complications, and though ARF is commonly encountered in the first days following earthquakes, it has excellent outcomes in cases where renal replacement treatment is available [39, 48]. Patient time spent trapped under debris, multiple CIs, male gender, presence of infection, and creatine kinase level all serve as predictors of ARF [19]. Based on these, missed compartment syndrome remains one of most common causes of malpractice lawsuits [23]. Vigilant prehospital emergency care is therefore crucial for reducing complications [39].

If not treated in a timely manner, CS has a very high mortality rate, with poor prognoses [2, 20, 26, 49]. In addition, CS has high morbidity and mortality even in cases when fluid therapy is administered; many CS patients develop severe electrolyte disorders, systemic inflammation, and multiple organ failures, including kidney dysfunction and cardiac failure, which are secondary to severe rhabdomyolysis and reperfusion injuries [17, 35, 42]. Furthermore, on-site CS mortality remains high due to the lack of effective drugs based on definite diagnoses [50]. Death is often the result of hypovolemia and hyperkalemia [19]. In particular, AKI is the leading cause of death as a result of CS [15]. There are many forms of cell death in AKI; a typical form is ferroptosis [15]. Many factors influence the outcomes of these injuries, and clinicians must understand IR injuries in order to minimize patient morbidity and mortality [22].

### Knowledge from actual disasters

Actual CS data exists, but is scarce [14]. Data is as follows:

In a validation of the medical records of 372 CI patients from the 1995 Kobe earthquake, the data was retrospectively analyzed, and risk factors were assessed for CS-related outcomes [51]. The results showed that two predictive triage models — initial evaluation in the field and secondary assessment in a hospital — could prove especially useful for helping non-disaster-experts distinguish earthquake victims with a high risk of severe CS from those who run a lower risk [51]. Applying this model could enable relief workers to make better use of limited medical and transportation resources following a disaster [51].

In 2003, an earthquake struck Bam, Iran, and a study was performed regarding the epidemiological aspects of the earthquake from a nephrological perspective [52]. There, a questionnaire was sent to the reference hospitals, and an analysis was performed on the resulting database of 2,086 patients hospitalized for trauma within the first 10 days [52]. The results showed that the hospitalized patients were primarily young and middle-aged adults [52]. ARF patients were generally entrapped longer, and hospitalized later and for longer periods [52]. The latter group had greater incidence rates of medical complications, surgical procedures, and mortality [52]. For preventing ARF and subsequent mortality in earthquake scenarios, early extrication and rapid hospitalization with appropriate multidisciplinary care proved to be vital [52].

In Haitian earthquake scenarios, CIs were a major cause of death, and as many as 25% of earthquake victims suffered from CS [48]. The incidence rate of CS-related ARF varied by earthquake intensity and amount of time spent under rubble, ranging from 0.5% to 25% [48]. More than half of patients with renal failure needed renal replacement therapy [48].

### Other future medical science concerns

With regard to diagnosis, there have been recent advances with newer pathophysiology concepts, accompanied by new diagnostic and therapeutic modalities [23]. These include the concept of using inflammatory mediators as markers, and the use of anti-inflammatories as medical adjunct therapy [23]. New diagnostic modalities now available include ultrafiltration catheters, near-infrared spectroscopy, and radio-frequency identification implants [23]. All of these aim to make up for current shortcomings in the diagnostic armamentarium available to trauma surgeons [23].

It has become clear that disaster relief as a whole must transition from “good intentions” and charity-based approaches to a professional, outcome-oriented form of response [53]. However, medicine as practiced in disaster- and conflict-stricken areas is effectively defined by environments in which disorder, unpredictability, and resource shortages are considered the norm, rather than an exception [53]. Many logistical problems related to patient treatment are a result of these chaotic circumstances; as a result, there is a need for medical and logistical recommendations on how to treat crush victims [18]. Bearing this consideration in mind, the World Health Organization (WHO; Geneva, Switzerland) and its partners have set out to improve disaster response systems [53]. In austere environments with limited resources and slow evacuation times, medics need to be prepared to identify trends and predict outcomes based on injury mechanisms and patient presentations [6]. More specifically, emergency services for disasters such as earthquakes should always be ready in terms of accurate registration; correct triage assignments; correct data entry; sufficient resources, teams, and equipment; and adequate treatment areas [54]. Further, it is vital to provide sufficient disaster training, prepare feasible disaster relief plans, and conduct regular drills [54]. The Emergency Medical Team (EMT) classification system resulting from this therefore requires common standards of care for teams that plan to handle disaster response in resource-constrained environments [53]. In order to clearly establish these standards, the WHO EMT Secretariat worked in collaboration with the International Committee of the Red Cross (ICRC; Geneva, Switzerland) and leading experts from other stakeholder non-governmental organizations, resulting in the production of a guide on properly managing limb injuries in disaster- and conflict-stricken areas [53]. These patients’ progression of care often depends on appropriate management at each EMT level of classification, as well as proper transfer between levels, depending on the resources available [13]. The management recommended by the WHO and ICRC at each EMT level of classification is shown in Additional file 1. However, as a result of the very limited epidemiological and quantitative data, there remains no standardized triage approach for earthquake victims [51, 55].

Given the occurrence and high mortality rate of CS patients, traditional treatments do not yet meet clinical needs; it is therefore necessary to develop efficient, convenient new treatments [2]. Due to CS’s pathophysiological mechanisms, the efficacy of new therapies has been shown in animal models of CS, particularly the rat model [2]. Details are as follows: first, skin damage provided a valid measure of trans-epidermal water loss and CS severity, suggesting that these models may prove useful for helping professionals inexperienced in disaster management to identify earthquake victims who run a high risk of severe CS [55]. Second, many new anti-inflammatory and anti-oxidant drug therapies have been found to be highly efficacious [2]. Some of these are expected to become specific drugs for emergency treatment of the large numbers of patients who could develop CS following future earthquakes, wars, and other disasters [2]. Third, free zinc ions played a critical immune system role: neutrophil function was impaired by zinc depletion, and zinc chelators contributed to resolving exacerbated inflammatory response and attenuated muscle breakdown in the acute phase following CS [27]. Therefore, attenuating the acute inflammatory reaction with zinc chelators could prove promising as a therapeutic strategy not only for CS, but for other inflammatory-reaction-driven diseases as well [27]. Fourth, the effects of salvianolic acid B include protection of the heart and kidney, as well as anti-oxidative, anti-inflammatory, anti-bacterial, and anti-apoptotic properties [16]. Administering salvianolic acid B led to significant survival improvements following CS, by decreasing dysfunction of the heart and kidneys, decreasing inflammation, and decreasing dysfunction of the endothelium through improved mitochondrial function, as well as through antibacterial effects by means of neutrophil extracellular trap systems [16]. Fifth, treatment with fluid containing 3-O-beta-d-xylopyranosyl-6-O-beta-d-glucopyranosyl-cycloastragenol (astragaloside-IV), isolated from *Astragalus membranaceus*, led to dramatic CS survival improvements due to direct and indirect anti-oxidative effects in the kidney, as well as improvements to dysfunction of the mitochondria and inflammation owing to the action of astragaloside-IV as a nitric oxide (NO) donor in the injured muscle [17]. Sixth, treatment using 200 μmol/kg sodium nitrite helps prevent IR-induced muscle damage by means of NO’s protective effects and the suppression of systemic inflammation, leading in turn to increased CS survival rates [56]. Seventh, carbon monoxide (CO)-enriched red blood cells (which can be prepared both at hospitals and at the sites of disasters) serve to dramatically suppress the pathogenesis of CS-related AKI, leading to improved mortality by suppressing renal injuries associated with heme protein [32]. CO-enriched red blood cells therefore have the potential to serve as a practical therapeutic agent against the disaster nephrology associated with CS [32]. Eighth, the pharmacokinetic characteristics of dexamethasone support the potential use of dexamethasone in medical care in disaster-stricken areas [57]. Ninth, anisodamine is widely used in China as a treatment for shock, and activating the α7 nicotinic acetylcholine receptor (α7nAChR) mediates this anti-shock effect [50]. Some of this work was designed to test whether activating α7nAChR with anisodamine decreased CS mortality shortly after decompression; the result showed that activating α7nAChR with anisodamine could decrease on-site CS mortality, due at least partially to the decline of serum potassium through the insulin signaling-Na/K-ATPase pathway [50]. On the other hand, one article reported a pre-clinical porcine model showing the same response to injury and treatment seen in human physiology, enabling the reliable testing of both surgical and non-surgical therapies for CS [31]. However, the current state of pre-clinical CS modeling is inadequate; the model should ideally replicate human disease [31].

Although there are few potential medical treatments of CS in clinical practice, and they need further study to ensure their safety in future clinical application, animal research into drug treatments for CS holds great significance for the future of effective early treatment of CS patients at the scene [2]. More importantly, following in-depth study of the aforementioned medical therapy, it is expected to be used in emergency situations, before or during decompression of injured patients at disaster sites [2].

## Conclusions

Prehospital providers and emergency clinicians who possess a comprehensive understanding of pathophysiology, diagnosis, management, and tactical considerations can optimize patient outcomes, and be prepared, given the tools at hand, for the progression of CI into CS. Given recent developments in what is considered possible, both technologically and surgically, this field is likely to see tremendous advances in the coming years, further emphasizing its importance and the need for continued research.

### Supplementary Information

**Additional file 1.** Recommended management at each EMT level of classification WHO and ICRC. The progress of CS patients’ care can often depend on appropriate management at each EMT classification level, as well as the proper transfer between levels, and depends on the resources available.

## Data Availability

Not applicable.

## Abbreviations

CS: Crush syndrome
CI: Crush injury
AKI: Acute kidney injury
ACS: Acute compartment syndrome
CT: Computed tomography
IR: Ischemia reperfusion
NaHCO_3_: Sodium bicarbonate
ARF: Acute renal failure
HBOT: Hyperbaric oxygen therapy
WHO: World Health Organization
EMT: Emergency Medical Team
ICRC: International Committee of the Red Cross
Astragaloside-IV: 3-O-beta-d-xylopyranosyl-6-O-beta-d-glucopyranosyl-cycloastragenol
NO: Nitric oxide
CO: Carbon monoxide
α7nAChR: α7 Nicotinic acetylcholine receptor

## References

[1] Long B, Liang SY, Gottlieb M (2023). Crush injury and syndrome: a review for emergency clinicians. Am J Emerg Med.

[2] Li N, Wang X, Wang P, Fan H, Hou S, Gong Y (2020). Emerging medical therapies in crush syndrome - progress report from basic sciences and potential future avenues. Ren Fail.

[3] Chavez LO, Leon M, Einav S, Varon J (2016). Beyond muscle destruction: a systematic review of rhabdomyolysis for clinical practice. Crit Care.

[4] Genthon A, Wilcox SR (2014). Crush syndrome: a case report and review of the literature. J Emerg Med.

[5] Strauss MB (2012). The effect of hyperbaric oxygen in crush injuries and skeletal muscle-compartment syndromes. Undersea Hyperb Med.

[6] Anderson JL, Cole M, Pannell D (2022). Management of severe crush injuries in austere environments: a special operations perspective. J Spec Oper Med.

[7] Bouklouch Y, Schmidt AH, Obremskey WT, Bernstein M, Gamburg N, Harvey EJ (2022). Big data insights into predictors of acute compartment syndrome. Injury.

[8] Wallin K, Nguyen H, Russell L, Lee DK (2016). Acute traumatic compartment syndrome in pediatric foot: a systematic review and case report. J Foot Ankle Surg.

[9] Cui H, Xiong G, Zhang C, Xiao Z (2018). Diagnosis and treatment of crush syndrome of chest and arm. Zhongguo Xiu Fu Chong Jian Wai Ke Za Zhi.

[10] Yang X, Tang N, Li L, Xu G, Dai J, Tao K, He C, Huangfu C (2022). Management of a patient with cardiac arrest, intestinal ischemia necrosis, multiple fractures, hemorrhagic shock, renal failure, disseminated intravascular coagulation, and thrombosis after severe abdominal crush injury: A case report. Exp Ther Med.

[11] Raniga SB, Mittal AK, Bernstein M, Skalski MR, Al-Hadidi AM (2019). Multidetector CT in Vascular Injuries Resulting from Pelvic Fractures: A Primer for Diagnostic Radiologists. Radiographics.

[12] Williamson M, Vanacore F, Hing C (2018). Pubic symphysis diastasis sustained from a waterslide injury. J Clin Orthop Trauma.

[13] Management of limb injuries during disasters and conflicts. https://extranet.who.int/emt/sites/default/files/_A%20Field%20Guide.pdf. Accessed by 2 April 2023.

[14] Altintepe L, Guney I, Tonbul Z, Türk S, Mazi M, Ağca E, Yeksan M (2007). Early and intensive fluid replacement prevents acute renal failure in the crush cases associated with spontaneous collapse of an apartment in Konya. Ren Fail.

[15] Qiao O, Wang X, Wang Y, Li N, Gong Y (2023). Ferroptosis in acute kidney injury following crush syndrome: a novel target for treatment. J Adv Res.

[16] Murata I, Sugai T, Murakawa Y, Miyamoto Y, Kobayashi J, Inoue Y, Kanamoto I (2022). Salvianolic acid B improves the survival rate, acute kidney dysfunction, inflammation and NETosis-mediated antibacterial action in a crush syndrome rat model. Exp Ther Med.

[17] Murata I, Abe Y, Yaginuma Y, Yodo K, Kamakari Y, Miyazaki Y, Baba D, Shinoda Y, Iwasaki T, Takahashi K, Kobayashi J, Inoue Y, Kanamoto I (2017). Astragaloside-IV prevents acute kidney injury and inflammation by normalizing muscular mitochondrial function associated with a nitric oxide protective mechanism in crush syndrome rats. Ann Intensive Care.

[18] Sever MS, Vanholder R (2013). Management of crush victims in mass disasters: highlights from recently published recommendations. Clin J Am Soc Nephrol.

[19] Wuthisuthimethawee P, Lindquist SJ, Sandler N, Clavisi O, Korin S, Watters D, Gruen RL (2015). Wound management in disaster settings. World J Surg.

[20] Goodman AD, Got CJ, Weiss AC (2017). Crush Injuries of the Hand. J Hand Surg Am.

[21] Kuznik BI (2013). Coagulation and fibrinolytic activity of lymph in various pathological conditions (review of own and literature data). Patol Fiziol Eksp Ter.

[22] Gillani S, Cao J, Suzuki T, Hak DJ (2012). The effect of ischemia reperfusion injury on skeletal muscle. Injury.

[23] Harvey EJ, Sanders DW, Shuler MS, Lawendy AR, Cole AL, Alqahtani SM, Schmidt AH (2012). What's new in acute compartment syndrome?. J Orthop Trauma.

[24] Strauss MB (2022). The role of hyperbaric oxygen for acute traumatic ischemias. Undersea Hyperb Med.

[25] Whiffin ANH, Spangler JD, Dhir K, Zhang R, Ferguson JD (2019). Bathroom Entrapment Leading to Cardiac Arrest From Crush Syndrome. Prehosp Emerg Care.

[26] Osuka A, Miyao D, Kuge Y, Nakajima S, Kuroki Y, Ueyama M (2021). Good recovery without decompression fasciotomy for crush syndrome caused by using a Japanese-style toilet. Trauma Case Rep.

[27] Haruta Y, Kobayakawa K, Saiwai H, Hata K, Tamaru T, Iura H, Ono G, Kitade K, Kijima K, Iida K, Kawaguchi K, Matsumoto Y, Kubota K, Maeda T, Konno DJ, Okada S, Nakashima Y (2022). Zinc chelator treatment in crush syndrome model mice attenuates ischemia-reperfusion-induced muscle injury due to suppressing of neutrophil infiltration. Sci Rep.

[28] Peiris D (2017). A historical perspective on crush syndrome: the clinical application of its pathogenesis, established by the study of wartime crush injuries. J Clin Pathol.

[29] Lutter C, Schöffl V, Hotfiel T, Simon M, Maffulli N (2019). Compartment Syndrome of the Foot: An Evidence-Based Review. J Foot Ankle Surg.

[30] Dodd A, Le I (2013). Foot compartment syndrome: diagnosis and management. J Am Acad Orthop Surg.

[31] Honjol Y, Monk R, Schupbach D, Merle G, Harvey EJ (2023). Porcine Model of Acute Compartment Syndrome. J Orthop Trauma J Orthop Trauma.

[32] Taguchi K, Ogaki S, Nagasaki T, Yanagisawa H, Nishida K, Maeda H, Enoki Y, Matsumoto K, Sekijima H, Ooi K, Ishima Y, Watanabe H, Fukagawa M, Otagiri M, Maruyama T (2020). Carbon Monoxide Rescues the Developmental Lethality of Experimental Rat Models of Rhabdomyolysis-Induced Acute Kidney Injury. J Pharmacol Exp Ther.

[33] Scharman EJ, Troutman WG (2013). Prevention of kidney injury following rhabdomyolysis: a systematic review. Ann Pharmacother.

[34] Schwartz DS, Weisner Z, Badar J (2015). Immediate Lower Extremity Tourniquet Application to Delay Onset of Reperfusion Injury after Prolonged Crush Injury. Prehosp Emerg Care.

[35] Murata I, Imanari M, Komiya M, Kobayashi J, Inoue Y, Kanamoto I (2020). Icing treatment in rats with crush syndrome can improve survival through reduction of potassium concentration and mitochondrial function disorder effect. Exp Ther Med.

[36] Harl MJ (2020). Defining the Role of Hyperbaric Oxygen Therapy as an Adjunct to Reconstructive Surgery. Surg Clin North Am.

[37] Dougherty JE (2013). The role of hyperbaric oxygen therapy in crush injuries. Crit Care Nurs Q.

[38] Lindenmann J, Smolle C, Kamolz LP, Smolle-Juettner FM, Graier WF (2021). Survey of Molecular Mechanisms of Hyperbaric Oxygen in Tissue Repair. Int J Mol Sci.

[39] Zhang X, Bai X, Zhou Q (2014). First-aid treatments of crush injuries after earthquake: 2 special cases. Am J Emerg Med.

[40] Gerdin M, Wladis A, von Schreeb J (2012). Surgical management of closed crush injury-induced compartment syndrome after earthquakes in resource-scarce settings. J Trauma Acute Care Surg.

[41] Laverdiere C, Montreuil J, Bouklouch Y, Lorange JP, Dion CA, Harvey EJ (2023). Predictors of foot acute compartment syndrome: big data analysis. J Foot Ankle Surg.

[42] Arango-Granados MC, Cruz Mendoza DF, Salcedo Cadavid AE, García Marín AF (2020). Amputation in crush syndrome: a case report. Int J Surg Case Rep.

[43] Del Piñal F (2020). An update on the management of severe crush injury to the forearm and hand. Clin Plast Surg.

[44] Del Piñal F, Urrutia E, Klich M (2017). Severe crush injury to the forearm and hand: the role of microsurgery. Clin Plast Surg.

[45] Nayar SK, Alcock HMF, Edwards DS (2022). Primary amputation versus limb salvage in upper limb major trauma: a systematic review. Eur J Orthop Surg Traumatol.

[46] Kumar RS, Singhi PK, Chidambaram M (2017). Are we justified doing salvage or amputation procedure based on mangled extremity severity score in mangled upper extremity injury. J Orthop Case Rep.

[47] Ahmad S, Anees M, Elahi I, Fazal-E-Mateen (2021). Rhabdomyolysis leading to acute kidney injury. J Coll Physicians Surg Pak..

[48] Bartal C, Zeller L, Miskin I, Sebbag G, Karp E, Grossman A, Engel A, Carter D, Kreiss Y (2011). Crush syndrome: saving more lives in disasters: lessons learned from the early-response phase in Haiti. Arch Intern Med.

[49] Jiang D, Zhao J, Wang X, Shao M, Gong H, Zhou F, Liu Y, Wang L (2020). Prevention strategies for traumatic cardiac arrest. Zhonghua Wei Zhong Bing Ji Jiu Yi Xue.

[50] Fan BS, Zhang EH, Wu M, Guo JM, Su DF, Liu X, Yu JG (2016). Activation of α7 nicotinic acetylcholine receptor decreases on-site mortality in crush syndrome through insulin signaling-Na/K-ATPase pathway. Front Pharmacol.

[51] Aoki N, Demsar J, Zupan B, Mozina M, Pretto EA, Oda J, Tanaka H, Sugimoto K, Yoshioka T, Fukui T (2007). Predictive model for estimating risk of crush syndrome: a data mining approach. J Trauma.

[52] Hatamizadeh P, Najafi I, Vanholder R, Rashid-Farokhi F, Sanadgol H, Seyrafian S, Mooraki A, Atabak S, Samimagham H, Pourfarziani V, Broumand B, Van Biesen W, Lameire N (2006). Epidemiologic aspects of the Bam earthquake in Iran: the nephrologic perspective. Am J Kidney Dis.

[53] Jensen G, Bar-On E, Wiedler JT, Hautz SC, Veen H, Kay AR, Norton I, Gosselin RA, von Schreeb J (2019). Improving management of limb injuries in disasters and conflicts. Prehosp Disaster Med.

[54] Uz İ, Çetin M, Songur Kodik M, Güvenç E, Karbek Akarca F, Ersel M (2022). Emergency department management after the 2020 Aegean Sea - Izmir earthquake. Ulus Travma Acil Cerrahi Derg.

[55] Murata I, Kawanishi R, Inoue S, Iwata M, Kobayashi J, Inoue Y, Kanamoto I (2019). A novel method to assess the severity and prognosis in crush syndrome by assessment of skin damage in hairless rats. Eur J Trauma Emerg Surg.

[56] Murata I, Miyake Y, Takahashi N, Suzuki R, Fujiwara T, Sato Y, Inoue Y, Kobayashi J, Kanamoto I (2017). Low-dose sodium nitrite fluid resuscitation prevents lethality from crush syndrome by improving nitric oxide consumption and preventing myoglobin cytotoxicity in kidney in a rat model. Shock.

[57] Murata I, Otsuka A, Hara C, Motohashi R, Kouno S, Inoue Y, Kanamoto I (2015). Pharmacokinetics characteristics of dexamethasone in crush syndrome model rats. Yakugaku Zasshi.

